# Managing Training Load in the Lead-Up to Competition: Session-Rating of Perceived Exertion, not the Acute-to-Chronic Workload Ratio, Associated with the Performance of Elite Swimmers

**DOI:** 10.5114/jhk/218950

**Published:** 2026-04-02

**Authors:** Alexanter Marinof, Jesús Martínez-Sobrino, Jesús Santos del Cerro, José María González-Ravé, Santiago Veiga

**Affiliations:** 1 Departamento de Deportes, Facultad de Ciencias de la Actividad Física y del Deporte (INEF), Universidad Politécnica de Madrid, Madrid, Spain.; 2 Department of Statistics, University of Castilla-La Mancha, Toledo, Spain.; 3 Sports Training Laboratory, Faculty of Sports Sciences, University of Castilla-La Mancha, Toledo, Spain.

**Keywords:** training monitoring, competitive swimming, performance, taper, strength and conditioning

## Abstract

This study aimed to investigate the dynamic relationship between training loads and swimming performance among elite swimmers over a 40-week training period. Training data from seven international-level swimmers (4 men and 3 women) were collected during three macrocycles comprising one target competition each. The best personal times at the beginning of the cycle (MT_p_) were used to calculate the change in the swimmer's performance at the end of each macrocycle (MT_1_, MT_2_, and MT_3_). The training volume in the pool and the gym, as well as the session rating of perceived exertion (_S_RPE) were used to calculate the acute-to-chronic workload ratio (ACWR). Associations between training loads and performance change were analyzed using linear mixed models (LMMs), with the swimmer identity being included as a random intercept to account for repeated measures. The higher accumulated dry-land and swimming load during the final seven weeks preceding competition was negatively associated with performance (z_D__S_RPE_kg7_: β = −20.3; z_W__S_RPE_km7_: β = −15.5), whereas ACWR-based models showed limited explanatory value. The findings of the study emphasize that monitoring the swimmer's _S_RPE in the lead-up to competition can be important for performance optimization.

## Introduction

Although swimming competitive events range from 50 m (for swims lasting 21–26 s) to 1,500 m (for swims lasting 15–17 min), it is a common practice in training programs around the world for swimmers to cover 10,000 to 20,000 m a day, six to seven days a week (Nugent et al., 2017), in addition to completing strength and conditioning sessions (dry-land) in weekly schedules (Wirth et al., 2022). The need for these high training volumes is justified for improving stroke mechanics efficiency within the aquatic environment (Sein et al., 2008), for adapting to cardiovascular and musculoskeletal endurance (González-Boto et al., 2008), and for developing mental toughness (Mujika and Padilla, 2003).

Within training programs, swimming sessions are integrated into the broader periodization framework, in which training content is systematically scheduled to optimize performance at key events (Mujika, 2017). The competitive season in swimming is typically divided into training blocks known as macrocycles, which, for elite swimmers, usually span approximately 15 weeks (Mujika, 2017) and include the preparation, competition, and recovery phases (Hellard et al., 2019; Mujika, 2017; Seiler, 2010). To achieve physiological responses during each macrocycle, the swimmer's workload is usually quantified as the external training load (ETL), including volume, intensity, and duration per session. The sessions' focus is oriented to different training zones (Wallace et al., 2009). According to Nugent et al. (2017), common swimming training zones include low-intensity training, threshold training, and high-intensity training. To facilitate effective training cycle programming, adaptations, and performance enhancement, coaches introduce a gradual increase in training load between macrocycles, with an average week-to-week variability of 6% (Hellard et al., 2019).

The specific contribution of the dry-land training load to competitive swimmers remains notably underreported, even though dry-land training constitutes an integral component of contemporary swimming preparation. Evidence suggests that dry-land training interventions can elicit meaningful improvements in swimming performance, particularly in sprint events, as demonstrated by significant gains in 50-m and 100-m performances, stroke frequency, and the stroke index (Lopes et al., 2020). Moreover, strength capacities relevant to the exercise prescription under maximal and submaximal loading conditions have been well described across multiple sports, including swimming. The training load associated with joint-specific adaptations has been comprehensively characterized (Dhahbi et al., 2024b, 2025). Nevertheless, although the performance benefits of dry-land training are increasingly recognized, the specific contribution of the dry-land ETL and the internal training load (ITL) to competitive swimming remains insufficiently understood.

Despite training programs often being designed around the ETL, physiological stress imposed on swimmers, known as the ITL, drives training adaptations (Wallace et al., 2009). Physiological indicators (such as the heart rate [HR], blood lactate and cortisol level profiles, and VO) and subjective measures (such as the rating of perceived exertion [RPE]) are considered ITL metrics (Borg et al., 1987; Impellizzeri et al., 2005). Foster et al. (2001) proposed a low-cost method known as the session RPE (_S_RPE), which can offer a practical approach for ITL evaluation. The method combines the RPE, using which athletes rate the intensity of the session, with the training volume and time. The _S_RPE method has been shown to correlate significantly with HR-based measures (*r* = 0.55–0.94) and training volume (*r* = 0.37–0.81) in swimming (Wallace et al., 2009), and indirect observations of its relationship with athletic performance have been recorded in team sports (Hurwitz et al., 2022). Finally, in the context of competitive swimming, the _S_RPE method is applied to limit the risks of overtraining and injuries (Collette et al., 2018; De Andrade Nogueira et al., 2016; Psycharakis, 2011).

A promising tool to evaluate the training load is the acute-to-chronic workload ratio (ACWR), calculated as the ratio of the training load from the previous week (acute workload) to the rolling average of the previous four weeks (chronic workload) (Gabbett, 2016). Gabbett and Whiteley (2017) reported that maintaining an optimal ACWR is critical, as high values (>1.5) or low (<0.8) are associated with an increased risk of injury and potential performance declines. A study on tennis reported no significant association between the ACWR and match outcomes, despite ACWR values being close to 1; this suggests that the relationship between workload balance and competitive performance may not be as straightforward as previously thought (Myers et al., 2021). Although swimming research has focused mainly on the use of the ACWR for injury prevention and management (Collette et al., 2018), its impact on performance remains less understood.

From an ETL-based perspective, tapering strategies characterized by an initial overload phase followed by a reduction in training volume while maintaining high intensity have been shown to enhance competitive performance (Mujika, 2017). Nevertheless, the relationship between ITL indicators, such as the _S_RPE and the ACWR, and competition performance in swimming remains unclear. Therefore, this study aimed to investigate the dynamic relationship between training load and swimming performance among elite swimmers, providing information on how ITL management can help competitive swimmers optimize performance during major competitive events.

## Methods

### Participants

Seven competitive swimmers (4 men and 3 women; body height: 179.0 ± 5.9 cm; body mass: 69.05 ± 5.05 kg; age: 19.97 ± 2.18 years; sum of 7 skin folds: 60.98 ± 22.37 mm) volunteered to participate in this study. All were members of the Spanish Senior National Team and had 6–8 years competitive experience, with their average personal best times reaching 803 ± 45 points, according to the World Aquatics points (WAPs). According to McKay et al.'s (2022) classification framework, these personal best times corresponded to a classification competitor of level 4 (international level). The study adhered to the Declaration of Helsinki guidelines and was approved by the Institutional Ethics Committee of the Facultad de Ciencias de la Actividad Física y del Deporte (INEF), Universidad Politécnica de Madrid, Madrid, Spain (protocol code: 20240207; approval date: 13 January 2025). For participants who were minors, written informed consent was obtained from their legal guardians and all of them and their coach provided written approval for retrospective analysis.

### Measures

**Figure 1:**
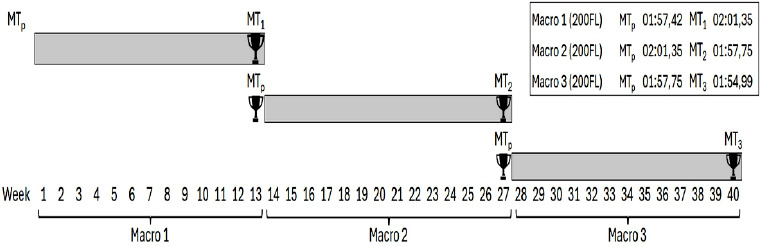
Illustration of a 40-week season structured into three macrocycles, each associated with the major competition.

Before this study began, each swimmer's best time in their main event during the major competition of the previous season (MT_p_) was collected and expressed as WAPs. Then, during a 40-week training period from the beginning to the end of the 2022–2023 season (September to July), swimmer training data were registered. The season included three macrocycles that covered three main competitions: the winter national championships, the spring national championships, and the summer national championships—all held in a 50-m pool, with the water temperature varying between 26.6 and 27.3°C (Figure 1). Swimmers typically completed nine in-water training sessions per week (2 h each) and four supervised dry-land training sessions per week (75 min each), following standardized team warm-up routines, nutritional guidance, and medical support under the supervision of experienced national-team staff. Training adherence was consistently high (>98%) for both in-water and dry-land sessions, with minimal inter-individual variability, ensuring uniform exposure to the prescribed training program. The menstrual cycle of the female participants was not monitored in the present study, and, given the small sample size, it was unlikely to influence the training load-performance relationships across a full competitive season.

The first macrocycle spanned a 13-week training block, the second covered a 14-week training block, and the third included a 13-week training block, with the main competitions held in the last week of the macrocycles. In these macrocycles, the performance of the swimmers in their main events was evaluated. These performances were quantified using WAPs and recorded as Meet Time (MT) 1, 2, or 3, respectively.

Following Foster et al.’s (2001) recommendations, participants received standard instructions to report their RPE within 30 min after each training session using the CR-10 scale (Borg et al., 1987). The volume of each training session per swimmer, both in water and during dry-land training, was quantified as time (in minutes) and expressed as distance (in km) for swimming sessions and load (in kg) for dry-land sessions, respectively, and recorded in an Excel sheet.

### Design and Procedures

A descriptive design of a collective case series, as previously used in a study on a group of athletes who underwent the same or similar training regimes over a certain period (Hellard et al., 2019), was used with the group of international-level swimmers. Although each swimmer followed a specific training program, the research design was primarily observational, as removing such training from a control group was neither ethical nor possible in such trained populations. Therefore, the training programs were designed and implemented by the coaches without the researchers' interference.

The ITL was quantified using the _S_RPE method, as described by Foster et al. (2001). According to this method, the _S_RPE was calculated by multiplying the RPE of the swimmer by the duration (in minutes) or volume (in km for swimming and kg for dry-land sessions) of each training session (Dhahbi et al., 2024a). The dry-land training load was quantified as the total lifted volume (in kg), calculated as the product of the load, repetitions, and sets for each exercise. This method does not reflect the relative intensity of the exercise performed by each swimmer (Dhahbi et al., 2024b). Consequently, this metric should be interpreted primarily as an individualized, practical indicator of the ITL rather than an absolute measure of training intensity. The results were quantified as the in-water training load in km (W__S_RPE_km_) and minutes (W__S_RPE_min_), and the dry-land training load variables in kg (D__S_RPE_kg_) and minutes (D__S_RPE_min_). Weekly _S_RPE values were determined by adding the _S_RPE values of all sessions in the week and reporting the final values in arbitrary units (AU). Wallace et al. (2009) validated this approach for swimming.

Furthermore, consistently with the Murray et al.'s (2016) study, the ACWR was used as an index of athlete’s training readiness, using exponentially weighted moving averages to compare training loads completed in a recent training block (seven days) with the chronic training load completed over a period of four weeks. Exponentially weighted moving averages were calculated using a decay factor defined as λ = 2 / (N + 1), where *N* represented the time window. A coupled ACWR approach was adopted, whereby the acute workload week was included in the chronic workload calculation. The moving average model was used to assign the athlete's earlier workloads with progressively diminishing weight compared to training sessions completed more recently, which represented a greater weight. This resulted in the following variables: the in-water and dry-land ACWR in minutes (W_ACWR_min_ and D_ACWR_min_, respectively), the in-water ACWR in km (W_ACWR_km_), and the dry-land ACWR in kg (D_ACWR_kg_). The change in performance (Δ) was quantified using WAPs as the difference between the swimmer's best time in the main competition of the current training cycle (MT) and their best time in the main competition of the previous training cycle (MT_p_).

### Statistical Analysis

Training and competitive data were statistically analyzed using R software (v.4.3.0 for Windows). Descriptive statistics, including mean ± SD values and dispersion coefficients, were calculated for all variables. According to the objectives of this study, the training load indicators (the _S_RPE and the ACWR) with the personal best times of the previous training cycle (MT_p_) were used to predict the change in swimmers’ performance at the end of each macrocycle (ΔMT_1_, ΔMT_2_, and ΔMT_3_). As a preliminary analysis, a correlation matrix for the training load indicators (W__S_RPE_km_, D__S_RPE_kg_, W__S_RPE_min_, D__S_RPE_min_, W_ACWR_km_, D_ACWR_kg_, W_ACWR_min_, and D_ACWR_min_) was built to check for multicollinearity in the performance regression models, as multicollinearity could cause some explanatory variables to be non-significant.

Then, given the repeated-measures structure of the data (three macrocycles per swimmer), linear mixed models (LMMs) were used to account for within-subject dependence. The swimmer’s identity was included as a random intercept, while training load indicators (the _S_RPE, the ACWR, and MT_p_) were included as fixed effects. All continuous predictors were *z*-standardized (e.g., z_W__S_RPE_km7_) before the analysis. This was done to capture physiologically meaningful changes related to each swimmer's typical training load while accounting for repeated observations of swimmers. This approach allowed the LMMs to distinguish the true relationships between training load and performance from stable between-swimmer differences, which might explain the discrepancies observed with simpler regression models. Based on tapering physiology and periodization literature (Hellard et al., 2019; Mujika and Padilla, 2003), training-load indicators were summarized over seven weeks before each target competition. This time window was selected prior to the lead-up competition period, during which the training load is typically reduced while intensity is maintained.

## Results

The evolution of the _S_RPE and the ACWR over 40 weeks for seven international-level swimmers is shown in Figures 2a–b. The mean W__S_RPE_km_ was 15.97 ± 3.77 AU in 40 weeks, reaching a peak of 24.23 AU in training week 7 and a minimum of 6.11 AU in training week 28. Notably, low values of D__S_RPE_kg_ and D__S_RPE_min_ were recorded during the competition weeks (13, 26, and 40). The average training load for W__S_RPE_min_ was 28.33 ± 6.31 AU, and the D__S_RPE_min_ training load averaged 6.54 ± 3.94 AU. For the ACWR variables, mean values were 1.00 ± 0.09 and 0.96 ± 0.12 for the swimming and dry-land training in minutes, respectively, while, for the kilometer and kilogram variables, mean values were 0.99 ± 0.09 and 0.91 ± 0.12, respectively. The W_ACWR_km_ and D_ACWR_kg_ showed maximum values during training weeks 7 and 10 (24.63 and 12.46 AU, respectively) and W_ACWR_min_ and D_ACWR_min_ during training weeks 30 and 2 (30.47 and 19.53 AU, respectively).

**Figure 2a:**
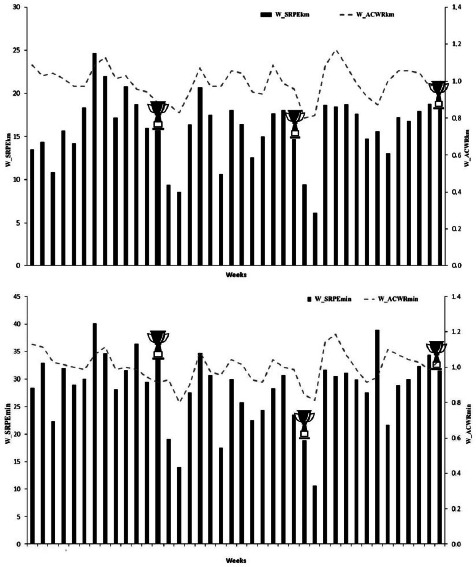
. Evolution of the session rating of perceived exertion (_S_RPE) and the acute-to-chronic workload ratio (ACWR) in water training for seven international-level swimmers over a 40-week season.

**Figures 2b:**
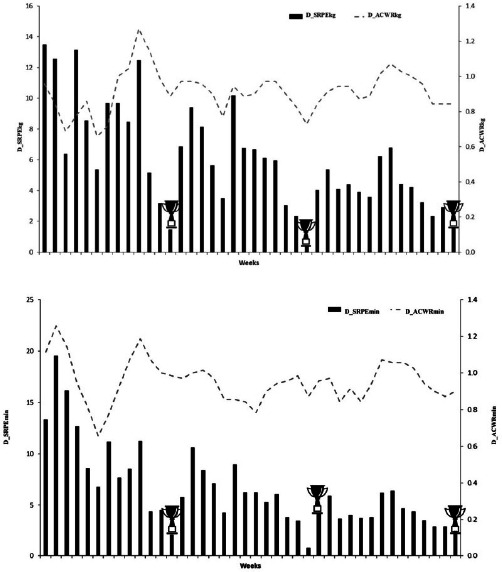
Evolution of the session rating of perceived exertion (_S_RPE) and the acute-to-chronic workload ratio (ACWR) in dry-land training for seven international-level swimmers over a 40-week season.

**Figure 3:**
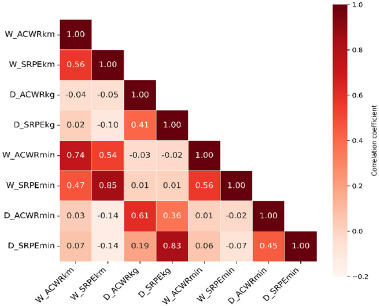
Relationship between training load indicators in water and on dry land training.

The four LMMs used to predict swimming performance are presented in Table 2 (a–d). Across models, the main distinction was the training load metric used for the analysis (the _S_RPE as opposed to the ACWR). In Model 2a, the _S_RPE expressed as z_W__S_RPE_km7_ and z_D__S_RPE_km7_ showed the most consistent and interpretable results. Better MT performance (in WAPs) was associated with better MT_p_, but negatively related to greater accumulated swimming and dry-land load during the final seven weeks (z_D_SRPE_kg7_: β = −20.3; z_W__S_RPE_km7_: β = −15.5). Simulation-based sensitivity analyses showed that the repeated-measures design had a high probability of detecting effects for key training load variables. Specifically, in Model 2a, sensitivity was high (98%) for the dry-land training load expressed as z_D_SRPE_km7_ and high for the swimming load expressed as z_W__S_RPE_km7_ (88%). These estimates are conditional on the observed effect sizes and should be interpreted as indicators of sensitivity rather than confirmatory statistical power. Model 2b, expressed as z_W__S_RPE_min7_ and z_D_SRPE_min7_, showed similar but weaker associations compared to the above. Higher dry-land and in-water training loads during the final seven weeks were negatively associated with competition performance (z_D_SRPE_min7_: β = −22.6; z_W__S_RPE_min7_: β = −16.7). Simulation-based sensitivity analyses indicated good sensitivity for the z_D__S_RPE_min7_ (87%), but only moderate sensitivity for the in-water z_W__S_RPE_min7_ (72%), suggesting that both associations were directionally consistent.

**Table 1:** Personal best times (PBs) and World Aquatics Points (WAPs) of the seven international-level swimmers included in this study.

Swimmer	Event	Gender	PB	WAP
1	200 m Butterfly	Male	1:54.99	883
2	400 m Individual Medley	Female	4:38.63	853
3	200 m Butterfly	Female	2:11.34	790
4	400 m Individual Medley	Male	4:19.32	831
5	200 m Backstroke	Female	2:17.01	729
6	200 m Butterfly	Male	1:57.42	829
7	100 m Backstroke	Male	0:56.27	760

Note: the applied time format is min:s.ms

**Table 2:** Linear mixed models (LMMs) examining the association between training load indicators (the session rating of perceived exertion [_S_RPE] and the acute-to-chronic workload ratio [ACWR], previous performance [MT_p_], and competition performance [World Aquatics Points]) across the final seven weeks before competition.

2a	Fixed effects			Random effects			
Coefficients	Estimate (β)	Std. Error	t-statistic	Groups	Name	Variance	Std. Dev.
Intercept	769.14	17.08	45.02	Swimmer Intercept 1959.8 44.27 Residual 249.3 15.79			
z_MTP	13.67	5.68	2.40				
z_D_SRPEkg7	−20.38	4.60	−4.43				
z_W_SRPEkm7	−15.53	4.49	−3.46				
z_b_ W_SRPEkm7	17.26	4.70	3.67				
z_b_D_SRPEkg7	−7.40	5.02	−1.47				
2b	Fixed effects			Random effects			
Coefficients	Estimate (β)	Std. Error	t-statistic	Groups	Name	Variance	Std. Dev.
(Intercept)	769.14	13.15	58.48	Swimmer Intercept 1075.5 32.79 Residual 405.1 20.13			
z_MTP	14.31	7.19	1.98				
z_D_SRPEmin7	−22.60	6.44	−3.50				
z_W_SRPEmin7	−16.70	5.84	−2.85				
z_b_W_SRPEmin7	18.73	6.32	2.96				
z_b_ D_SRPEmin7	0.15	5.56	0.02				
2c	Fixed effects			Random effects			
Coefficients	Estimate (β)	Std. Error	t-statistic	Groups	Name	Variance	Std. Dev.
(Intercept)	769.14	5.81	132.25	Swimmer Intercept 0.0 0.00 Residual 710.2 26.65			
z_MTP	42.14	6.64	6.34				
z_D_ACWRkg7	11.12	7.64	1.45				
z_W_ACWRkm7	−34.96	7.76	−4.50				
z_b_W_ACWRkm7	13.73	6.77	2.02				
z_b_D_ACWRkg7	−3.43	6.88	−0.49				
2d	Fixed effects			Random effects			
Coefficients	Estimate (β)	Std. Error	t-statistic	Groups	Name	Variance	Std. Dev.
(Intercept)	769.14	7.99	96.15	Swimmer Intercept 0.0 0.00 Residual 1344.0 36.65			
z_MTP	51.03	9.60	5.31				
z_D_ACWRmin7	−1.27	8.62	−0.14				
z_W_ACWRmin7	−14.95	8.73	−1.71				
z_b_W_ACWRmin7	5.34	9.54	0.56				
z_b_D_ACWRmin7	−8.27	9.06	−0.91				
				

Note. All predictors were z-standardized; therefore, β coefficients represent the expected change in performance (World Aquatics points [WAPs]) for a single standard deviation change in the predictor within the same swimmer. Variables labeled z_ indicate standardized training load or performance values averaged over the final seven weeks before the competition, while variables labeled b_ represent the linear slope (the rate of change) of the corresponding training load across these seven weeks. The models were fitted using LMMs, with the swimmer included as a random effect

ACWR models 2c and 2d were less stable, showing singular fits and limited explanatory value, particularly when the ACWR was expressed as W_ACWR_min_ and D_ACWR_min_. Overall, an excessive ACWR in the final weeks before competition impaired performance.

## Discussion

This study investigated the dynamics between the ITL and swimming performance in international-level swimmers over a 40-week training cycle. According to this study's results, the training load during the seven weeks before the competition was the most related to competition performance. Avoiding increases in training load indicators, both in swimming and dry-land sessions, during the seven weeks leading to competition may promote favorable performance outcomes, highlighting the importance of carefully managing the swimming ITL through the _S_RPE when approaching key competitions.

### Session Rating of Perceived Exertion (_S_RPE) and Performance

Training load indicators (D__S_RPE_kg_ and W__S_RPE_km_) throughout the 40 weeks peaked approximately six weeks before major competitions (weeks 7–10), yielding 12.46 and 24.63 AU, respectively. These peak loads aligned with linear periodization models that progressively built workloads before a taper phase to enhance performance (Mujika and Padilla, 2003). In the weeks immediately prior to the competition (13, 26, and 40), a clear decrease in training load was observed that could be associated with an improved race-day performance (Hellard et al., 2013; Mujika and Padilla, 2003; Seiler, 2010). In fact, during the last four weeks of cycles 1, 2, and 3, W__S_RPE_km_ progressively decreased to 50.0%, 53.3%, and 50.0%, respectively. Similarly, D__S_RPE_kg_ decreased by 54.8%, 38.1%, and 48.2% in cycles 1, 2, and 3. Mujika and Padilla (2003) reported that a significant 50–90% reduction in training volume and training frequency in the lead-up to a competition can induce 0.5–6% improvements in personal best times in cyclical sports. Changes in performance in the present study comprised a 2.3% decrement in cycle 1, but 2.3% and 0.2% improvements in cycles 2 and 3, respectively. This could reflect a lower importance in the competition phase for cycle 1 compared to cycles 2 and 3. At the beginning of the swimming season, swimmers were changing from general to more specific preparation, and race-specific adaptations probably did not occur. Similar patterns have been reported in cyclical sports (Mujika and Padilla, 2003) and swimming (Hellard et al., 2019; Mujika et al., 2002), with better results generally seen later in the season.

During the final seven weeks leading to competition, the LMM analysis showed high levels of simulation-based sensitivity and significant associations between training load indicators and swimming performance. The higher swimming training load expressed as z_W__S_RPE_km7_ was negatively associated with competition performance (β = −15.5), showing a greater simulation-based sensitivity than the time-based indicator (z_W__S_RPE_min7_). This statement is in line with established tapering frameworks in swimming (Hellard et al., 2019; Mujika and Padilla, 2003). In particular, the _S_RPE is widely used as a practical indicator of the ITL and has been shown to align well with training intensity (García-Ramos et al., 2014) and with heart rate-derived measures and overall training volume (Wallace et al., 2009). However, the present results are the first to associate the swimming ITL with performance of elite, competitive swimmers.

For the dry-land variables, a possible association was observed between z_D__S_RPE_kg7_ (β = −20.3) and competition performance, suggesting that excessive dry-land training in the final weeks may compromise readiness. Accordingly, reducing the dry-land training load during this period could enhance neuromuscular recovery and facilitate performance gains, thereby ensuring optimal performance in water (Wirth et al., 2022). This represents an important and novel finding of the present study, as no previous reports on competitive swimmers have examined the combined influence of in-water and dry-land training loads on competition performance. This is so despite dry-land training being a consistent component of elite swimming programs. Nevertheless, it is important to emphasize that dry-land training is not detrimental to competition performance. Experimental evidence demonstrates that well-designed, specific strength interventions can improve torque production and correct muscular imbalances (Agrebi et al., 2024). Therefore, the observed associations likely reflect excessive or poorly timed loading rather than the inherent value of strength training.

### ACWR and Performance

The weekly ACWR values in the present study (W_ACWR_km_: 0.99 ± 0.09; D_ACWR_kg_: 0.91 ± 0.12) were maintained within the established “safe” range of 0.8 to 1.3 (Gabbett and Whiteley, 2017; MacMillan et al., 2020) and showed standard deviations consistent with the 10–15% variation that Jones et al. (2016) proposed. The highest values of W_ACWR_km_ (1.07) and D_ACWR_kg_ (1.27) were recorded in weeks 7 and 10 prior to the major competition, respectively. These values during the last seven weeks before the competition indicated that the weekly and monthly training loads were consistent and that the drop in training load was not drastic enough to accelerate the downside of the ratio. This was aligned with the guidelines for reducing swimming training volume while maintaining intensity to maintain an effective training stimulus and adequate fitness levels (Hellard et al., 2019; Mujika and Padilla, 2003) and to maintain the ACWR in the preferred range. However, the models, including the ACWR (Table 2c–2d), did not demonstrate stability, showing singular fits and limited explanatory value, particularly when the ACWR was expressed as W_ACWR_min_. These findings agree with previous reports on tennis in which longitudinal analyses of junior players showed no significant association between the internal or external ACWR and match outcomes. Although ACWR markers were maintained close to 1, they did not predict match results, suggesting that a balanced workload may be insufficient to explain competitive performance (Myers et al., 2021). This study's results indicated that when ACWR values were close to 1, they were not independently associated with MT performance, suggesting that the ACWR alone may be insufficient to explain the performance outcome in the context of overall training load. It is crucial to understand that the ACWR was primarily used in swimming as an injury management index (Collette et al., 2018) and should not be interpreted merely as a training load metric, but rather as an index of fatigue reflecting the balance between recent and chronic load (Gabbett, 2016; Hulin et al., 2013). The misapplication of W_ACWR_km_ as a direct prescription for tapering volume reductions could potentially lead to suboptimal performance outcomes.

### Relationship between MTp and Performance

In elite sport, the initial performance level constrains the magnitude of achievable improvement (Costa et al., 2010). For instance, Del Castillo et al. (2022) report for elite swimmers that performance gains over one training cycle can predict swimming success in the following cycle (R^2^ = 0.68–0.73). Therefore, the role of previous performance MT_p_ as a predictor of the competition outcome is statistically and physiologically expected among international-level swimmers. Under these conditions, training-load variables are expected to exert a comparatively smaller influence on performance. However, by modeling performance change between consecutive training cycles (ΔMT) rather than MT_p_, the present analysis substantially reduced the confounding effect of stable expertise-related factors, allowing for the clearer identification of training-load effects. Within this framework, this study's findings indicate that MT_p_ is not merely a retrospective marker, but a good predictor of an athlete's adaptive response to training (Table 2). This underscores the need for coaches to consider athletes' baseline performance when prescribing training loads, as a higher initial capacity may facilitate greater physiological adaptations (Allen et al., 2014; Mujika et al., 2002).

### Practical Implications and Limitations

According to this study, integrating W__S_RPE_km_ and D__S_RPE_kg_ into training assessments and session programming can allow coaches to manage the ITL and recovery more effectively, particularly during competitive phases. From an applied perspective, during the seven weeks before a major competition, a gradual reduction in W__S_RPE_km_ with the clear moderation of D__S_RPE_kg_ appears to be beneficial to competition outcomes. In addition, monitoring the ACWR could be useful for describing training load stability; however, this should not be interpreted as a direct measure of training load or competition readiness. Therefore, rather than targeting specific ACWR thresholds for swimming and dry-land training, coaches may benefit more from identifying trends in load management and supporting optimal performance timing. Moreover, the timing of _S_RPE peaks relative to competition phases should be carefully managed to align with tapering goals and maximize performance outcomes. Personalized training plans that reflect past performance, set incremental goals, and incorporate intermediate competitions can boost athletes' confidence and create positive momentum.

Future research should aim to replicate this study with swimmers across different events and distance specializations, allowing for a deeper, swimming-specific analysis of how training load metrics interact to influence performance. To further advance athletic performance optimization, contemporary approaches that go beyond traditional volume- and intensity-based paradigms, such as machine learning and AI-driven methods, could enhance personalized training prescriptions and support injury prevention in sports such as swimming (Souaifi et al., 2025).

## Conclusions

In conclusion, this study underscores the critical importance of managing the ITL both in the water and on dry land for elite-level swimmers through specific metrics, such as W__S_RPE_km_ and D__S_RPE_kg_, to optimize swimming performance. The findings indicate that thorough regulation of the ITL induced by training programs, particularly in the seven weeks leading to major competitions, can induce enhanced performance outcomes. Excessive D__S_RPE_kg_ and W__S_RPE_km_ seven weeks before major competitions may detrimentally affect swimming performance, highlighting the necessity for a balanced approach between the ITL and recovery. By integrating dry-land and in water ITL indicators, coaches can refine training plans to reinforce recovery and optimize competition readiness. Additionally, the predictive value of personal best times (MT_p_) emphasizes the significance of customizing training programs to reflect individual progress and event-specific requirements.
